# Targeted activation of androgen receptor signaling in the periosteum improves bone fracture repair

**DOI:** 10.1038/s41419-022-04595-1

**Published:** 2022-02-08

**Authors:** Kuo-Chung Lan, Kuo-Ting Wei, Pei-Wen Lin, Ching-Chen Lin, Pei-Ling Won, Ya-Fen Liu, Yun-Ju Chen, Bi-Hua Cheng, Tien-Min G. Chu, Jia-Feng Chen, Ko-En Huang, Chawnshang Chang, Hong-Yo Kang

**Affiliations:** 1 Center for Hormone and Reproductive Medicine Research, Department of Obstetrics and Gynecology, Kaohsiung Chang-Gung Memorial Hospital and Chang Gung University, College of Medicine, Kaohsiung, Taiwan; 2 Graduate Institute of Clinical Medical Sciences, Chang Gung University, College of Medicine, Kaohsiung, Taiwan; 3 Department of Clinical Pathology, Kaohsiung Armed Forces General Hospital, Kaohsiung, Taiwan; 4 Department of Restorative Dentistry, Indiana University School of Dentistry, Indianapolis, IN USA; 5 Division of Rheumatology, Allergy and Immunology, Department of Internal Medicine, Kaohsiung Chang-Gung Memorial Hospital and Chang Gung University, College of Medicine, Kaohsiung, Taiwan; 6 George Whipple Lab for Cancer Research, Departments of Pathology and Urology, University of Rochester, Rochester, NY USA

**Keywords:** Bone development, Metabolic bone disease

## Abstract

Low testosterone level is an independent predictor of osteoporotic fracture in elderly men as well as increased fracture risk in men undergoing androgen deprivation. Androgens and androgen receptor (AR) actions are essential for bone development and homeostasis but their linkage to fracture repair remains unclear. Here we found that AR is highly expressed in the periosteum cells and is co-localized with a mesenchymal progenitor cell marker, paired-related homeobox protein 1 (Prrx1), during bone fracture repair. Mice lacking the *AR* gene in the periosteum expressing Prrx1-cre (AR^-/Y^;Prrx1::Cre) but not in the chondrocytes (AR^-/Y^;Col-2::Cre) exhibits reduced callus size and new bone volume. Gene expression data analysis revealed that the expression of several collagens, integrins and cell adhesion molecules were downregulated in periosteum-derived progenitor cells (PDCs) from AR^-/Y^;Prrx1::Cre mice. Mechanistically, androgens-AR signaling activates the AR/ARA55/FAK complex and induces the collagen-integrin α2β1 gene expression that is required for promoting the AR-mediated PDCs migration. Using mouse cortical-defect and femoral graft transplantation models, we proved that elimination of AR in periosteum of host mice impairs fracture healing, regardless of AR existence of transplanted donor graft. While testosterone implanted scaffolds failed to complete callus bridging across the fracture gap in AR^-/Y^;Prrx1::Cre mice, cell-based transplantation using DPCs re-expressing AR could lead to rescue bone repair. In conclusion, targeting androgen/AR axis in the periosteum may provide a novel therapy approach to improve fracture healing.

## Introduction

Bone repair after fracture is a complex process in which the periosteum is stimulated to form a cartilaginous callus, which subsequently undergoes bone regeneration [1]. Removal or damage of the periosteum dramatically delays the healing of bone fractures [2]. Periosteal cells are a major source of soft callus in bone fracture [3], and a large proportion of cartilage and woven bone in the early fracture callus is derived from the periosteum adjacent to the fracture site [4]. The periosteum plays a key role in the repair and regeneration of fractures through the activation of multipotent periosteum-derived progenitor cells (PDCs) [5, 6]. Understanding how PDCs are activated, recruited, and migrate to the injury site, provides insight into the underlying mechanisms and is important for identifying approaches for improving fracture repair outcomes.

Androgens—sex steroids that are well documented to act as anabolic hormones in males—exert effects on skeletal development and homeostasis [7, 8]. Androgens act mainly through binding to the androgen receptor (AR), which functions as a ligand-inducible transcription factor that controls an integrated gene-expression program and nongenotropic signaling required for bone health and disease [9–11]. Men with complete androgen insensitivity syndrome owing to loss of function mutations in AR gene have diminished vertebral and femoral bone density [12], supporting a seminal role for the AR in the accrual and/or maintenance of bone density. Furthermore, androgens are critical for increased periosteal bone formation during puberty and aging in men [13], likely accounting for the lower incidence of spine fractures in men than women [14–16]. Notable in this context, a low serum level of free testosterone is an independent predictor of bone mineral density (BMD) and the risk of osteoporotic fracture in elderly men [17–19]. The data from observational studies with over 50,000 participants found that androgen deprivation therapy either through gonadotropin-releasing hormone agonist or orchiectomy increased the relative risk of fracture by 1.54 and 1.45, respectively [19–21], suggesting deficiency of androgen-AR signaling pathway increased fracture risk in men undergoing androgen deprivation.

Using genetically modified mouse models for targeted disruption of AR gene, we and others have shown that AR is essential for bone mineralization [22, 23] and deficiency of AR signaling in bone marrow mesenchymal stem/progenitor cells (MSCs) leads to increased adipogenesis, decreased osteogenic differentiation, and subsequent age-related bone loss [24, 25]. While male mice with targeted deletion of AR gene in osteoprogenitors [26], mature osteoblasts [27] or osteocytes [28] only have lower cancellous bone mass, but no the cortical phenotype, the role for AR in cortical fracture repair remains to be determined. Here, we sought to investigate whether the actions of androgen/AR signaling in PDCs are required for fracture repair.

## Results

### AR in the periosteum is required for regulation of callus volume and new bone formation during fracture healing

**Fig. 1:**
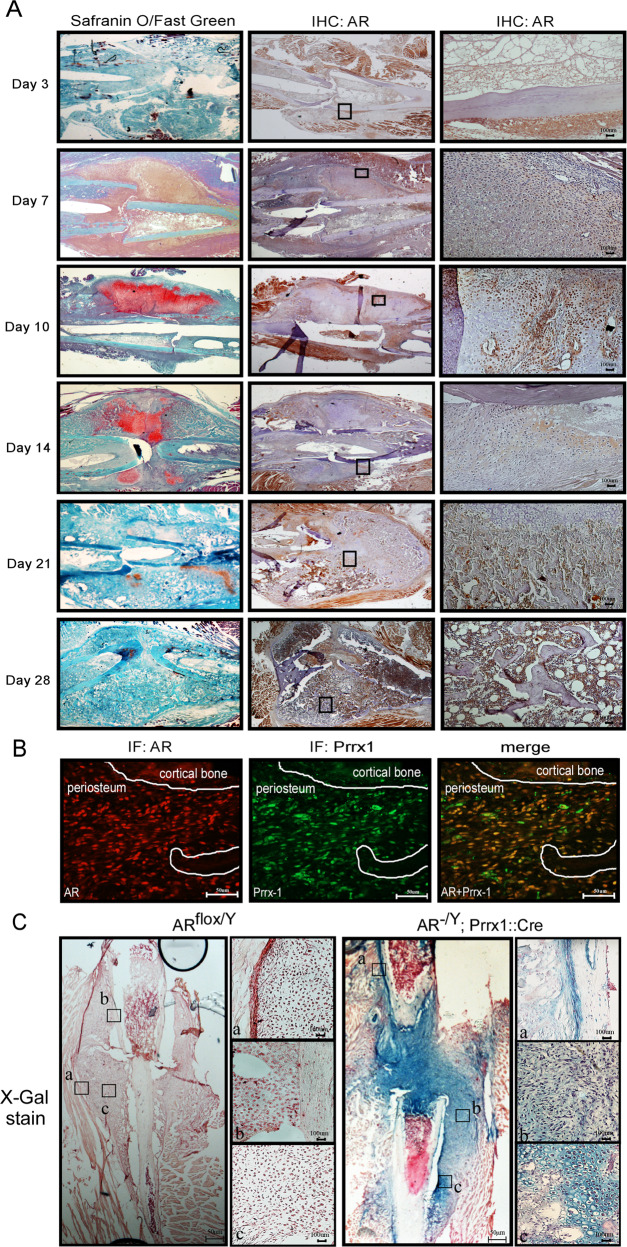
The AR is expressed in the periosteum during the fracture healing process. **A** Histological sections of post-fracture calluses at 3, 7, 10, 14, 21 and 28 days were analyzed by Safranin O/Fast Green staining and immunohistochemical analysis of AR expression. Scale bar = 100 µm. **B** Double-immunofluorescence staining revealed co-localization of the AR (red) and Prrx1 (green) in the periosteum of post-fracture calluses at 10 days. Scale bar = 50 µm. **C** X-gal–stained cells were observed in the post-fracture callus at 10 days in AR^-/Y^;Prrx1::Cre::Rosa26-LacZ mice (right panel); and AR^flox/Y^ mice (left panel) were used as negative controls. Scale bar = 50 µm. a. periosteum; b. mesenchyme; c. chondrocytes. Scale bar = 100 µm.

To study where the functional roles for AR signaling execute during fracture repair, we first analyzed the expression of the AR in the fracture callus by immunohistochemical staining. The results revealed that on days 3–7 post fracture, expression of the AR became evident in the periosteum, reaching a maximum on day 10 post fracture in the osteochondro progenitor cells of the inner cambium layer but not in the calcified chondrocytes from the cartilage of fracture callus (Fig. 1A). Paired-related homeobox protein 1 (Prrx1) has been detected in osteochondro progenitor cells in the periosteum at the fracture site as a periosteal marker [29]. Our immunofluorescence staining further showed the co-localized expression of the AR and Prrx1 in periosteal cells overlying the fracture callus at 10 days after fracture (Fig. 1B). To further clarify the potential of the periosteum as a source of cells for AR-mediated bone repair, we then established Prrx1-specific ARKO (AR^-/Y^;Prrx1;Cre-Rosa26-LacZ) mice. Expression of the *Rosa26-LacZ* reporter allele represents the Cre recombinase activity on the deletion of *AR* gene was further analyzed by X-gal staining in a model of femoral fracture. The presence of X-gal–positive cells revealed where recombination was observed in cells of the periosteum, undifferentiated cells at the fracture site and chondrocytes in the cartilaginous callus of Prrx1-specific ARKO mice but not control mice (Fig. 1C).

**Fig. 2:**
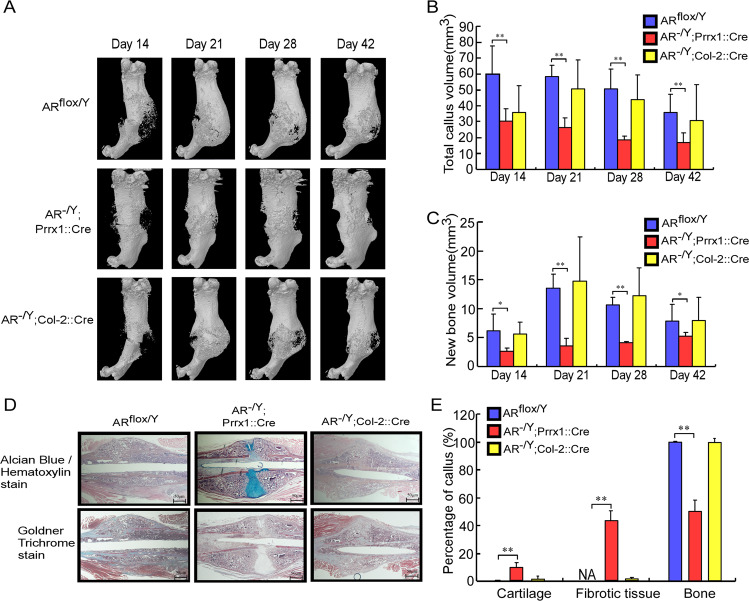
Prrx1-specific AR knockout (AR^-/Y^;Prrx1::Cre) mice have decreased callus volume and new bone volume in the fracture callus during fracture repair. **A** Representative images of micro-CT 3D reconstructions of the fracture site 14–42 days after fracture in AR^flox/Y^, AR^-/Y^;Prrx1::Cre and AR^-/Y^;Col-2::Cre mice. **B**, **C** Mineralized callus formation in AR^flox/Y^, AR^-/Y^;Prrx1::Cre and AR^-/Y^;Col-2::Cre mice, determined by quantitative analysis of total callus bone volume and new bone volume. **D** After micro-CT analysis, the cross-sections of 21-day fracture calluses were stained with Alcian Blue /Hematoxylin and Goldner Trichrome stain. Scale bar = 50 µm. **E** The percentages of cartilage, fibrotic tissue, and bone were quantified. Data are presented as mean ± SEM (*n* ≥ 3; *P < 0.05, **P < 0.001 compared with AR^flox/Y^ mice, one-way ANOVA).

To dissect the actions of AR signaling in a range of cell types that contribute to the process of fracture healing, we generated genetically engineered mice in which the *AR* gene was specifically ablated in periosteal cells (AR^-/Y^;Prrx1::Cre mice) or chondrocytes (AR^-/Y^;Col-2::Cre mice), and created a closed transverse femoral fracture to compare the bone healing with that in AR^flox/Y^ mice. As shown in the three-dimensional (3D) micro-computed tomography (micro-CT) images and quantitative analyses, fracture healing was delayed in AR^-/Y^;Prrx1::Cre mice, but not in AR^-/Y^;Col-2::Cre mice, compared with AR^flox/Y^ mice (Fig. 2A). Total callus volume (TV) (Fig. 2B) and new bone volume (BV) (Fig. 2C) in the fracture callus were significantly decreased in AR^-/Y^;Prrx1::Cre mice compared with AR^flox/Y^ mice on days 14–42 post fracture. A mix of bone, cartilage, and fibrotic tissue filled 21-day fracture calluses, as demonstrated using Alcian Blue/Hematoxylin (cartilage in blue color) and Goldner trichrome (bone in green color) staining (Fig. 2D). Quantitative histomorphometric analyses revealed that a lower percentage of new bone formation but a higher proportion of cartilage and fibrotic tissue were exhibited in the callus of AR^-/Y^;Prrx1::Cre mice than AR^flox/Y^ and AR^-/Y^;Col-2::Cre mice (Fig. 2E).

### AR signaling may play positive roles to increases osteogenic differentiation of PDCs in vitro

To elucidate the molecular pathways by which AR signaling regulates activation of periosteal PDCs during fracture repair, we first isolated and characterized PDCs derived from the callus periosteum of 9- to 11-day-old AR^flox/Y^ mice. As shown in Supplementary Fig. 1A, these isolated cells expressed several well-known markers for MSCs such as Sca-I, CD29, CD105 and CD140, but not the hematopoietic cell markers, CD11b, CD34 and CD45 similar to mouse D1 bone stromal precursor cells (Supplementary Fig. 1A). Using a Rosa-Cre mouse model that allows efficient deletion of the *AR* gene, we were able to isolate PDCs from both AR^flox/Y^ and AR^-/Y^;Prrx1::Cre mice. In contrast to the expression pattern that of the TM4 Sertoli cell line, these Rosa-positive PDCs expressed periostin, a key regulator of periosteal cells with high bone regenerative potential in the periosteum and their stem cell niche [30], as well as Prrx1, fibroblast growth factor receptor 3 and fibroblast growth factor 18, markers of periosteal cells to coordinate chondrogenesis and osteogenesis [29], suggesting that these PDCs have the osteochondrogenic potential (Supplementary Fig. 1B). To determine the multiple lineage differentiation capacity of these cells in vitro, we treated the cells with lineage-specific differentiation media and found that these cells are capable of differentiating into osteoblasts, adipocytes and chondrocytes, establishing the multipotency of these PDCs (Supplementary Fig. 1C, D). To determine whether AR signaling has osteogenesis-promoting effects on these PDCs, we cultured AR-overexpressing PDCs in osteogenic medium and measured cell mineralization by Von Kossa staining. As shown in Supplementary Fig. 1E, calcium deposition and expression of the small integrin-binding ligand N-linked glycoprotein gene family members including bone sialoprotein, matrix extracellular phosphoglycoprotein, and dentin matrix acidic phosphoprotein 1, which are important in AR-mediated matrix mineralization [22], were increased in PDCs stably overexpressing a constitutively active AR.

### Identification of AR downstream genes during bone fracture repair

Next, a microarray analysis was used to identify specific genes that were differentially expressed in PDCs isolated from AR^flox/Y^ and AR^-/Y^;Prrx1::Cre mice, particularly those with a role in bone fracture repair. Genes involved in the bone fracture repair processes, including those encoding extracellular matrix (ECM) proteins and cell adhesion molecules, as well as proteins that influence skeletal development, bone mineral metabolism, cell growth and differentiation, were selected (Supplementary Table 1). Notably, among the downregulated genes in PDCs isolated from AR^-/Y^;Prrx1::Cre mice were many members of the collagen family, including *Col1a1, Col1a2, Col2a1, Col6a1, Col6a2, Col7a1, Col11a1* and *Col12a1*, which have previously been described as important ECM proteins in fracture repair models [31, 32]. In addition, genes involved in cell–cell adhesion and cell-matrix adhesion, such as the integrins, *Itga2, Itga2b, Itgb1* and *Itga3*, also exhibited an overall reduction in expression in PDCs isolated from AR^-/Y^;Prrx1::Cre mice compared with AR^flox/Y^ mice (Supplementary Table 1). Results from RT-qPCR assay confirmed that mRNA levels of *AR, Col1a1, Col1a2, Col2a1, Col6a1, Col6a2, Col7a1, Col11a1, Col12a1, Itga2* and *Itgb1* were decreased in primary PDCs from AR^-/Y^;Prrx1::Cre mice (Supplementary Fig. 2).

### Collagens-integrins signaling is essential for AR to increase the cell migration and adhesion in PDCs

**Fig. 3:**
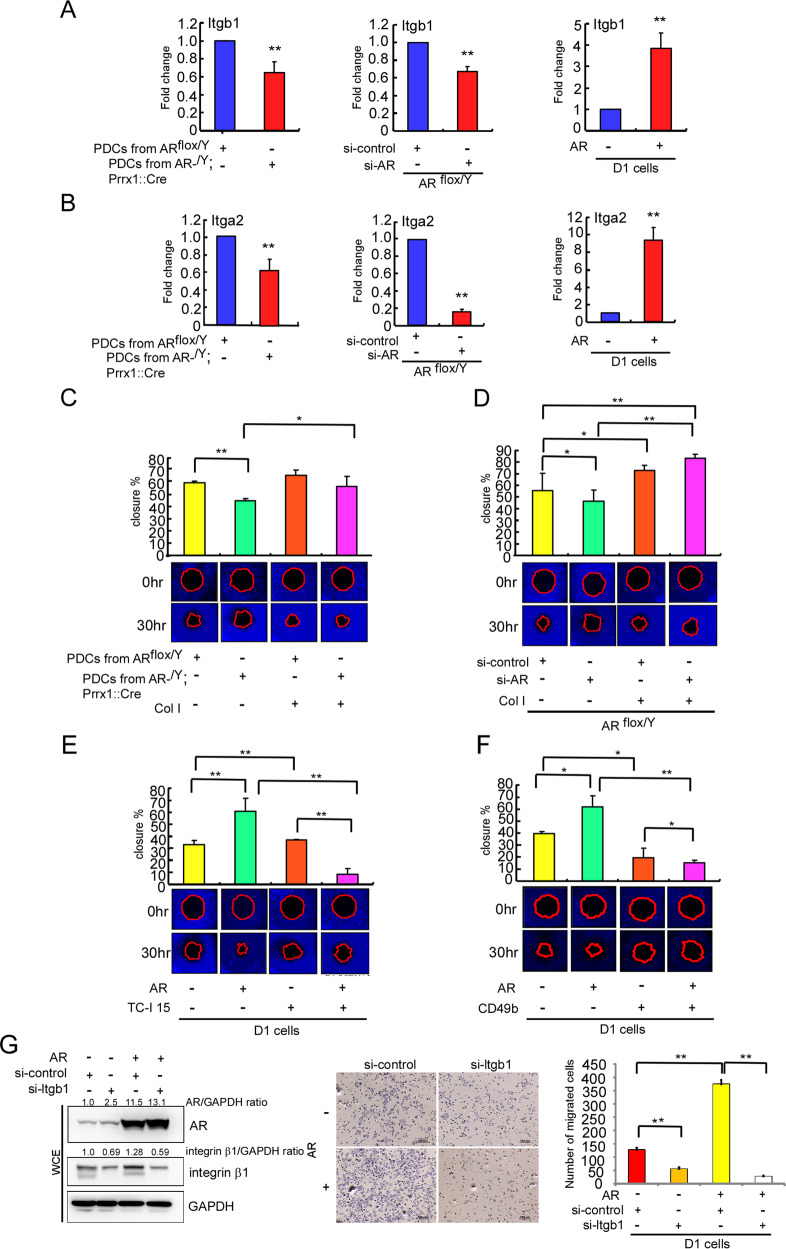
Collagens-integrins signaling is essential for AR to enhance the migration potential of PDCs in vitro. Primary PDCs were isolated from AR^flox/Y^ and AR^-/Y^;Prrx1::Cre mice. The AR in PDCs from AR^flox/Y^ mice was knocked down using AR-targeted siRNA (si-AR). D1 cells were transfected with Flag (pcDNA3-flag) or Flag-tagged AR (pcDNA3-flag-AR). **A**, **B** Itgb1 and Itga2 mRNA levels were quantified by RT-qPCR. **C**, **D** Cells were cultured with or without the integrin ligand, Col I. **E**, **F** Cells were incubated with or without the integrin α2β1 inhibitor TC-I or integrin α2β1 blocking antibody CD49b. (C–F) Migration capacity was measured using the Oris^TM^ Cell Migration Assay (CMA) kit. **G** D1 cells were transfected with pSG5 or AR (pSG5-AR). The integrin β1 in the transfected cells was knocked down using Itgb1-targeted siRNA (si-Itgb1). Cell migration assay was stained with Giemsa stain. AR, integrin β1 and GAPDH protein levels were then determined by western blot analysis. Experiments were conducted three times. Data were expressed as the mean ± SD. Statistical correlation of data was checked for significance by Student’s *t* test. *P < 0.05, **P < 0.001 was considered to indicate a statistically significance result.

Integrins are collagens-binding receptors expressed in bone and cartilage tissues, and serve bridges for cell–cell and ECM interactions during bone fracture repair [33–35]. We first applied the RT-qPCR assay to confirm that expression of the integrin genes, *Itga2* (encoding integrin α2) and *Itgb1* (encoding integrin β1), identified by our microarray, were suppressed in both AR^-/Y^;Prrx1::Cre PDCs and AR-knockdown AR^flox/Y^ PDCs, whereas re-expression of the AR increased Itgb1 and Itga2 mRNA levels in D1 cells (Fig. 3A, B).

The mobilization of adult skeletal stem cells/progenitors to allow deposition of cartilage and formation of bone at the injury site are key in promoting fracture repair [6]. Accordingly, we further studied the impact of AR signaling in the migration of primary PDCs and the mouse D1 bone marrow stromal precursor cell line. Results from a cell-migration analysis revealed that migration of primary PDCs from AR^-/Y^;Prrx1::Cre mice was decreased compared with that of PDCs from AR^flox/Y^ mice (Fig. 3C, left panel). Similar results were obtained when AR was knocked down by small interfering RNA (siRNA) in PDCs from AR^flox/Y^ mice, showing cell migration was also significantly decreased compared with cells transfected with control (scrambled) siRNA (Fig. 3D, left panel). In contrast, increase AR via adding AR-cDNA in D1 cells led to increase cell migration compared with control cells transfected with empty vector (Fig. 3E, F, left panel). These results were further confirmed by adding dihydrotestosterone (DHT) that led to an increase in the AR expression as well as the cell adhesion affinity and migration in a concentration-dependent manner, and these increased effects were reversed/blocked after adding antiandrogen hydroxyflutamide (Supplementary Fig. 3A–C). Similar results were also obtained when we replaced androgen/antiandrogen with AR-cDNA/AR-siRNA in D1 cells, showing cell adhesion and migration were positively regulated by androgen/AR (Supplementary Fig. 3D, E).

We then examined the impacts of the α2β1 integrin, a major receptor for collagen I, on AR- promoted cell migration. Adding collagen I led to reverse the decreased cell migration seen in AR^-/Y^;Prrx1::Cre PDCs (Fig. 3C, right panel) and AR-siRNA knockdown AR^flox/Y^ PDCs (Fig. 3D, right panel). In contrast, when we applied TC-I 15, the α2β1 integrin inhibitor, and CD49b neutralizing antibody for integrin α2 to suppress the collagen1-α2β1 integrin interaction, AR-promoted D1 cell migration was significantly decreased (Fig. 3E, F, right panel). Similar results were further confirmed when integrin-β1 was knocked down by integrin-β1-siRNA in D1 cells, showing AR-increased cell migration was also significantly decreased compared with cells transfected with control (scrambled) siRNA (Fig. 3G).

### AR/ARA55/FAK complex is required for the FAK activation to increase the androgen-mediated cell migration in D1 cells

**Fig. 4:**
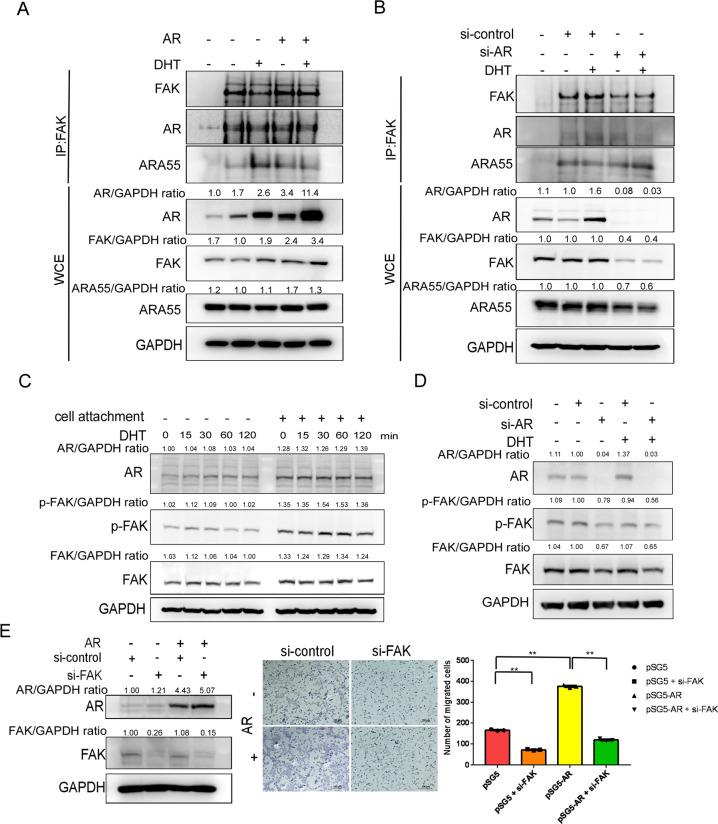
AR interacts with FAK/ARA55 complexes and FAK is required for AR-mediated cell migration. **A**, **B** AR-overexpressing D1 cells (pcDNA3-flag-AR transfection) and AR-knockdown D1 cells (si-AR) were treated with 10 nM DHT for 15 min. FAK, AR, ARA55 and β-tubulin protein levels were then determined by western blot analysis. Cell lysates were also immunoprecipitated (IP) with anti-AR or anti-FAK antibodies, after which immunoprecipitates were analyzed for interacting proteins (FAK, AR, ARA55) by western blotting. **C** D1 cells were exposed to 10 nM DHT for 15–120 min with or without the attachment. Phosphorylation of FAK at Tyr-397 (p-FAK PY397), total FAK and AR were assayed by western blot analysis. GAPDH was used as a loading control. **D** D1 cells were knocked down using si-AR and exposed to 10 nM DHT. Phosphorylation of FAK at Tyr-397 (p-FAK PY397), total FAK and AR were assayed by western blot analysis. GAPDH was used as a loading control. **E** D1 cells were transfected with pSG5 or AR (pSG5-AR). The FAK in the transfected cells was knocked down using FAK-targeted siRNA (si-FAK). Cell migration assay was stained with Giemsa stain. Experiments were conducted three times. Data were expressed as the mean ± SD. Statistical correlation of data was checked for significance by Student’s *t* test. *P < 0.05, **P < 0.001 was considered to indicate a statistically significance result. AR, FAK and GAPDH protein levels were then determined by western blot analysis.

Integrins couple the ECM to the cytoskeleton through the formation of cell adhesion complexes by regulation of the FAK-Hic-5/ARA55 axis during the cell migration [36]. To further study if nongenotropic AR signaling may function via activating the FAK-ARA55 function to increase cell adhesion and migration, we first used an immunoprecipitation approach to isolate the AR-interacting proteins, and we examined the recruitment of FAK and ARA55 to the AR protein complex upon DHT treatment. Whole-cell extract analysis revealed that DHT led to an increase in the protein expression of AR but not FAK or ARA55 (Fig. 4A, bottom panel). Immunoprecipitation assay demonstrated that the formation of AR/AR55/FAK complexes is not DHT-dependent (Fig. 4A, upper panel), and the interaction between AR and FAK was diminished via adding AR-siRNA (Fig. 4B, upper panel). Similar results were also obtained when we isolated and analyzed the FAK protein complex (Fig. 4A, B, middle panel).

Since changes in the cytoskeleton architecture and tyrosine phosphorylation of FAK proteins at focal contact sites are hallmarks of migrating cells [37], we then analyzed the effect of androgens-AR on tyrosine phosphorylation of FAK. DHT treatment for 15 or 60 min resulted in increasing the phosphorylation of FAK at Tyr-397 (Fig. 4C) and overexpressing AR led to an increase in the tyrosine phosphorylation of FAK (Fig. 4D). When FAK was knocked down by FAK-siRNA in D1 cells, AR-promoted cell migration was significantly decreased compared with cells transfected with control (scrambled) siRNA (Fig. 4E).

### Using a live segmental defect and bone graft transplantation models to prove delivery of testosterone and AR-expressing PDCs enhances bone repair

**Fig. 5:**
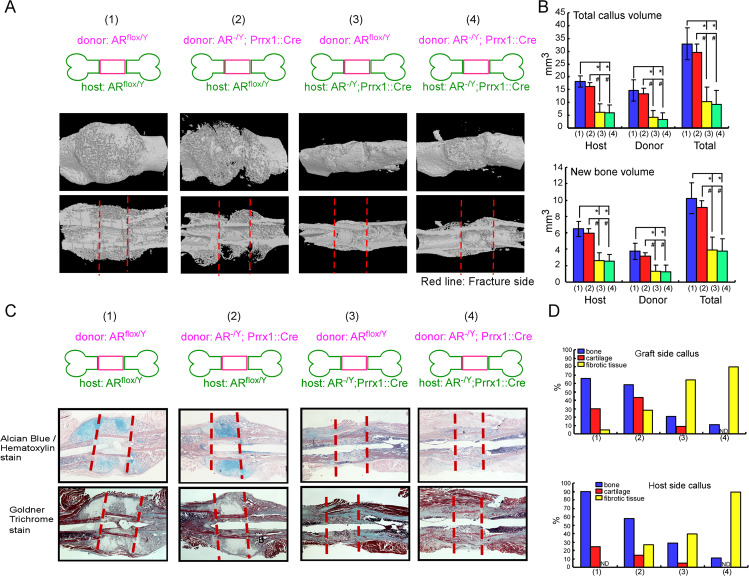
Femoral bone graft transplantations showed that deletion of AR in Prrx1-cre expressing cells from host mice impairs the function of AR^flox/Y^ donor graft. AR^flox/Y^ and AR^-/Y^;Prrx1::Cre host mice received transplantations of bone grafts from AR^flox/Y^ or AR^-/Y^;Prrx1::Cre donor mice and samples were acquired on 14 days after the operation. **A** Representative 3D micro-CT images of femoral bone graft transplantation between AR^flox/Y^ (WT, wildtype) and AR^-/Y^;Prrx1::Cre (ARKO) mice are shown: (1) WT graft to WT host, (2) KO graft to WT host, (3) WT graft to KO host, (4) KO graft to KO host. **B** Quantitative micro-CT analyses of total callus volume and new bone volume on host bone graft, donor bone graft and total bone segments are shown. Data are presented as mean ± SEM (*n* ≥ 5; *P < 0.001, ^#^*P* < 0.001, one-way ANOVA). **C** Representative images of cross-sections of 14-day fracture calluses from four different groups stained with Alcian Blue/Hematoxylin and Goldner Trichrome stain. Scale bar = 50 µm. **D** Histomorphometric analyses of callus were performed on histologic sections prepared from four groups of transplantations. The percent area of bone (blue bar), cartilage (red bar) and fibrotic tissue (yellow bar) on the graft side (upper panel) and host side (lower panel) were quantified.

To dissect the role of AR in the initiation of periosteal healing, we first utilized a murine femoral graft transplantation model, in which live segmental grafts from the same strains were transplanted and donor versus host cell involvement in healing was assessed. Micro-CT 3D reconstructions and quantitative analyses demonstrated abundant new bone formation along with the increasing callus volume of the graft in AR^flox/Y^-to-AR^flox/Y^ transplantations, whereas new BV and callus volume was significantly reduced in AR^-/Y^;Prrx1::Cre-to-AR^-/Y^;Prrx1::Cre transplantations compared to AR^flox/Y^-to-AR^flox/Y^ transplantation. When AR^flox/Y^ donor graft was transplanted into an AR^-/Y^;Prrx1::Cre host mice, a similar reduction of bone and callus formation was observed. In contrast, transplantation of AR^-/Y^;Prrx1::Cre graft into AR^flox/Y^ host mice resulted in the marked recovery of new bone and callus formation on both host bone and donor graft, suggesting that AR deficient donor cells were capable to form new bone when placed in AR^flox/Y^ host mice. (Fig. 5A, B). Quantitative histomorphometric analyses showed that AR^flox/Y^-to-AR^flox/Y^ transplantation resulted in normal endochondral bone healing with induction of periosteal bone and cartilage formation in the callus. In contrast, transplantation of AR^-/Y^;Prrx1::Cre graft into AR^-/Y^;Prrx1::Cre host mice led to a significant reduction of bone formation and the callus was occupied by the predominant proportion of fibrotic tissues but no cartilage formation. When an AR^-/Y^;Prrx1::Cre graft was transplanted into AR^flox/Y^ host mice, periosteal cell-initiated endochondral bone formation was not significantly altered. In contrast, transplantation of AR^flox/Y^ graft into an AR^-/Y^;Prrx1::Cre host mice resulted in the reduction of bone formation and a large amount of fibrotic tissues were found at the cortical junctions and along bone graft surface, similar to AR^-/Y^;Prrx1::Cre-to-AR^-/Y^;Prrx1::Cre transplantation, indicating that deletion of AR in Prrx1-cre expressing cells from host mice impaired the function of AR^flox/Y^ donor graft (Fig. 5C, D).

**Fig. 6:**
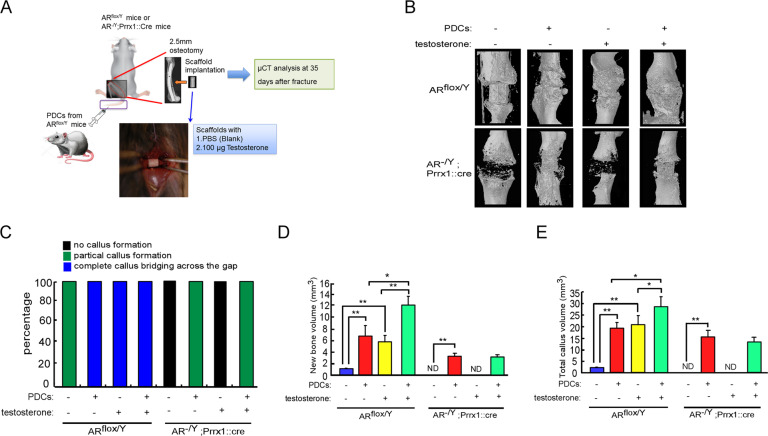
Combination of androgen therapy with the DPCs cell-based transplantation augments the repair of segmental bone defects. AR^flox/Y^ and AR^-/Y^;Prrx1::Cre host mice received transplantations of bone grafts from AR^flox/Y^ or AR^-/Y^;Prrx1::Cre donor mice and samples were acquired on 14 days after the operation. **A** AR^flox/Y^ and AR^-/Y^;Prrx1::Cre mice received segmental bone defects, and then were implanted with scaffold containing vehicle control or 100 μg testosterone. These host mice were also treated with PDCs from AR^flox/Y^ donor mice by tail vein injection. **B** 3D micro-CT images of defect sites are shown at 35 days post operation. **C** The percentages of no callus formation, partial callus formation and complete callus bridging across the gap were quantified by X-ray image analysis. **D**, **E** The new bone volume and total callus volume were quantified by micro-CT analysis. Data are presented as mean ± SEM (*n* ≥ 3; *P < 0.05, **P < 0.001, one-way ANOVA). ND not detected.

To investigate whether AR signaling in PDCs promotes bone fracture healing, we isolated primary PDCs from AR^flox/Y^ or AR^-/Y^;Prrx1::Cre mice. These donor PDCs were then injected via the tail vein into AR^flox/Y^ host mice with stable closed fracture, and TV and new BV were analyzed by micro-CT analysis during fracture repair (Supplementary Fig. 4A). The results revealed that PDCs derived from AR^-/Y^;Prrx1::Cre mice resulted in a significantly reduced amount of callus and bone with retaining a large port of unmineralized cartilages compared with AR^flox/Y^ mice-derived PDCs (Supplementary Fig. 4B–E). In contrast, re-expressing AR in PDCs derived from AR^-/Y^;Prrx1::Cre mice increased the TV and new trabecular bone formation (Supplementary Fig. 4B–E). To further evaluate potential strategies for experimental treatments in a segmental bone defects model, we created a 2.5-mm-wide osteotomy and implanted testosterone scaffolds in AR^flox/Y^ and AR^-/Y^;Prrx1::Cre mice in combination with or without injection of PDCs (Fig. 6A). The results from Micro-CT 3D reconstructions and quantitative analysis showed that implanted scaffolds containing testosterone increased TV, new bone formation and completed callus bridging across the fracture gap in AR^flox/Y^ mice, but not in AR^-/Y^;Prrx1::Cre mice (Fig. 6B–E), suggesting that AR is required for testosterone therapy to enhance callus volume and new bone formation. Notably, injected PDCs from AR^flox/Y^ mice also increased callus volume and new bone formation not only in both AR^flox/Y^ but also in AR^-/Y^;Prrx1::Cre mice (Fig. 6D, E). However, combination therapy with testosterone and PDCs formed a solid completed callus bridge (Fig. 6C) and had additional effects on new bone formation and callus volume in AR^flox/Y^ mice but not in AR^-/Y^;Prrx1::Cre mice (Fig. 6D, E).

## Discussion

**Fig. 7:**
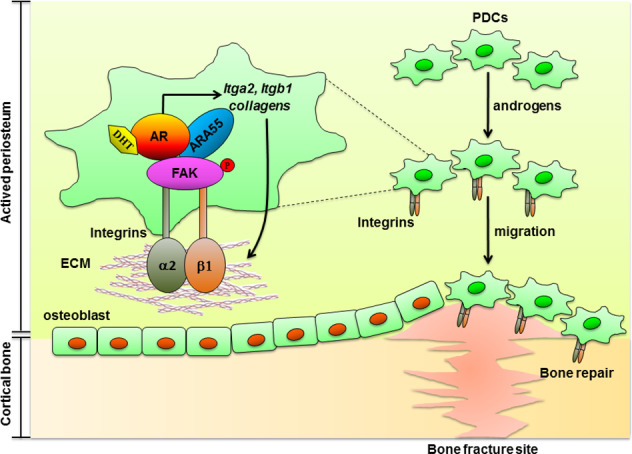
Novel androgen-AR-targeted approaches that promote homing of PDCs to bone formation and repair. The targeted androgens-AR and collagen-integrin α2β1 in PDCs can increase the formation of AR/ARA55/FAK complex that resulted promote the PDCs migration via activation of signaling mediated by ECM–integrin interactions, an effect that translates into increased periosteal bone formation and improved bone fracture repair. AR androgen receptor, ARA55 AR-associated protein 55, FAK focal adhesion kinase, ECM extracellular matrix, PDCs periosteum-derived progenitor cells.

The periosteum contains multipotent PDCs that improve bone regeneration being suitable for mimetic autograft design [38]. In this study, we elucidate the novel genomic and nongenomic AR actions on PDCs to accelerate bone callus formation in mouse femoral defect models via promoting collagen-integrin expression and interaction to activate AR/ARA55/FAK axis, therefore increasing the transduction of androgen signaling to focal adhesion complex in regulating PDCs migrated to the site of skeletal injury (Fig. 7).

Testosterone accelerates fracture healing in eugonadal male and female mouse models [39] and exerts a direct peripheral effect on the callus cells and stimulates fracture healing [40]. Androgens stimulate periosteal bone formation in both men and male rodents [41, 42] and are known to have a direct effect on bone healing, as it induced callus formation in segmental bone defect mouse models [43], which are in line with our present results that male mice lacking AR (AR^-/Y^;Prrx1::Cre) have decreased periosteal cell activity with low levels of collagens and integrins and testosterone can only promote bone fracture repair in AR^flox/Y^ control mice, but not in AR^-/Y^;Prrx1::Cre mice. Together, these data suggest androgen/AR activation in the cells of periosteum is required for the development of trabecular bone and periosteal bone formation in the calluses during endochondral ossification of bone repair.

The ECM serves as a reservoir for hormones, growth factors, and cytokines that regulate cellular activation and behavior. The main constituent of the ECM in bone is type I collagen [44]. Cell adhesion to extracellular collagens is mediated by a subset of integrins, which have a special collagen-binding domain [33, 45, 46]. Members of the β1 subfamily of integrins are reported to be the most highly expressed integrins and the predominant mediators of cell adhesion in osteoprogenitors and osteoblasts [47, 48]. Several studies have indicated that the interaction of α2β1 integrin with collagen I is a crucial signal for osteoblastic differentiation and matrix mineralization [49–52]. Integrins have been shown to express in many mesenchymal cells and are required for homing stem progenitor cells to fracture sites for bone repair [53]. Diminished callus size and cartilage synthesis was observed in α1β1 integrin-deficient mice during bone fracture healing [34]. The downstream signaling of ECM–integrin interactions is governed by FAK activation, which leads to induction of the mitogen-activated protein kinases ERK1/2 and PI3K–Akt signaling in osteoblasts [48]. Interaction between integrin and collagen I activates FAK, which is involved in osteoblastic differentiation and matrix mineralization [52, 54, 55]. Previous reports have shown that FAK activation through phosphorylation of Tyr-397 is critical for osteoblast adhesion and migration, as well as bone formation in fracture healing [56–58]. During the differentiation process, FAK is continuously activated from MSCs to osteoblasts [59, 60]. A FAK deficiency in osteoblasts and osteocytes in vivo results in delayed bone healing and remodeling [61, 62]. ARA55 is a homologous focal adhesion adaptor protein that coordinates cytoskeletal rearrangements in response to integrin signaling in focal adhesion. ARA55 also acts as an AR coregulator [63] to regulate AR target genes, affected androgen-mediated cell mobility [64]. It is possible that androgen/AR signaling first acquires ARA55 as a transcriptional coactivator to increase the expression of various collagens and their receptors to maintain the balance of ECM–integrin interactions, which may then activate the FAK via increasing the formation of focal adhesion complex to promote the migration of PDCs.

Bone autografting is the most effective grafting application [65], because such grafts contain bone-forming stem cells and proteins in the periosteum, which provide a framework and microenvironment for new bone growth during fracture healing. Removal of the periosteum from bone grafts dramatically decreased neovascularization and new bone and cartilage formation, leading to poor bone healing [4, 66]. Our current study showed that bone graft healing was more efficient in AR^flox/Y^ but not AR^-/Y^ host mice regardless of implanting with either AR^flox/Y^ or AR^-/Y^;Prrx1::Cre donor bone, suggesting that AR may orchestrate crucial healing signaling for activation and differentiation of periosteal progenitors in host mice and elimination of AR in periosteum of host mice at the early stage of fracture healing could lead to detrimental effects on periosteum-initiated cortical bone repair. Another notable outcome of our study was the success of bone regeneration by transplanted AR-expressing PDCs in a mouse femoral defect reconstruction model, illustrating the remarkable ability of AR-expressing PDCs to promote bone regeneration as an emerging concept in fracture therapy. However, how androgen signals dictate the cells from the periosteal progenitor lineage to be recruited and enriched to the injury sites of bone fracture, and whether androgen/AR signaling can activate the paracrine signalings of these cells in bone healing niches, remains to be further elucidated.

Incorporation of growth factors into the scaffold biomaterial to elicit the periosteal response can improve osteogenesis and angiogenesis in the fracture sites [67]. The most notable known signaling molecules are bone morphogenic proteins (BMPs), fibroblast growth factors, platelet-derived growth factor, vascular endothelial growth factor, transforming growth factor-β and insulin-like growth factors, which have positive effects on bone healing [68–71]. Similar to our finding of the temporal and spatial expression of AR during the early phase of fracture healing, BMP-2 and its receptors was also able to induce the interaction of α2β1 integrin with collagen I [50], in the callus on the periosteal surface of the femur just a few days after cortical bone fracture [72] and collaboratively works with testosterone to improve bone healing [43]. It is possible that bone healing was more efficient in AR^flox/Y^ but not AR^-/Y^ mice due to the presence of AR for the activation of BMP-2 and other growth factors signaling to recruit the DPCs which increase bone regeneration. However, detailed studies are needed to determine the crosstalk among BMP-2, other growth factors signaling and androgens/AR-promoted bone healing in the periosteum and whether androgens/AR pathway can be activated to improve the allograft efficiency in clinical fracture therapy.

In conclusion, we demonstrated that AR in DPCs is a positive regulator of bone repair, suggesting that androgen-treated PDCs represent a novel potential clinical implement for bone formation and regeneration. Therapeutic efficacy and reliability are two major advantages of bone graft implantation in repairing bone defects. If the very encouraging outcomes via increasing androgen/AR axis in the periosteum prove to be reproducible in mammals, it may pave the way for the development of clinical applications.

## Methods

### Animals

Detailed procedures for the generation of floxed AR mice and genotyping are described in our previous paper [73]. The strains of mosaic founder mice were C57BL/6 and 129Sv and backcrossed to C57BL/6J for 12 generations. Prx1-Cre (Prrx1::Cre) (Stock 005584), Col-2-Cre (Col-2::Cre) (Stock 003554) were purchased from The Jackson Laboratory. Eight-week-old male AR^flox/Y^ and AR knockout (AR^-/Y^;Prrx1::Cre and AR^-/Y^;Col-2::Cre) mice were used in this study. All mice were maintained on a C57BL/6 background throughout the study.

### Bone scaffolds

Scaffolds were produced as described previously [74]. Briefly, a thermal-curable polypropylene fumarate (PPF)/tricalcium phosphate (TCP) suspension was prepared by mixing PPF, N-vinyl pyrrolidinone, and TCP at a weight ratio of 1:0.75:0.66. The PPF/TCP slurry was then mixed with 0.5% benzoyl peroxide (thermal initiator) and 10 ml of dimethyl p-toluidine (accelerator), and cast into a wax mold to produce tube-shaped structures (outer diameter, 2 mm, inner diameter 0.6 mm, height 2.5 mm) with two 0.4 mm diameter side holes. These scaffolds were load bearing, as they allowed mobility of fractured bone when applied [74]. In this study, AR^flox/Y^ and AR knockout (AR^-/Y^;Prrx1::Cre) are required 14 days after the operation.

### Stable closed femoral fracture model

Eight-week-old mice were anesthetized with xylazine (10 mg/kg) and ketamine (80 mg/kg). An incision in the skin and underlying soft tissues lateral to the patellar tendon was made, and the tendon was displaced medially, after which a small hole was drilled into the distal femur using a 25-gauge needle. A stylus pin from a 25-gauge Quincke Type spinal needle was inserted into the intramedullary canal and clipped, after which the wound was sutured. Fractures were created by using a three-point bending design. Mice were given buprenorphine (0.1 mg/kg) for pain relief for 7 consecutive days, and then were returned to their home cages and allowed to move freely.

### Micro-CT and radiography

After the closure of the surgical wound in each mouse with a segmental defect, the left femur with a scaffold of each mouse at days 7, 14, 21, 28, and 42 was scanned to quantitate callus formation by micro-CT (Skyscan 1076). The right femur without scaffold was similarly examined to serve as a control. The scanned images were developed using an X-ray tube voltage of 45 kV and current 100 μA with a 0.5-mm aluminum filter. X-ray image slices were reconstructed using the NRecon (v.1.4.4; Skyscan) software system and the 3D micro-CT images and parameters of bone microarchitecture were calculated using CTAn (v.1.7.0.0; Skyscan) and CTvol (v.1.11.0.1; Skyscan) software. The parameters measured 3D reconstruction and quantitative analyses included the percentage of new BV and TV; trabecular thickness, number, and separation; structure model index; cortical BMD, thickness, and degree of anisotropy were analyzed for each femur according to our previous study [43].

### Femoral bone graft transplantations

Eight-week-old AR^flox/Y^ and AR^-/Y^;Prrx1::Cre host mice were anesthetized with xylazine (10 mg/kg) and ketamine (80 mg/kg). A 7–8-mm-long incision was made and the midshaft femur was exposed through blunt dissecting muscles without disturbing the periosteum. The 4-mm mid-diaphyseal backbone segment was removed from the femur of the host mice by cutting the bone using a saw bone. A 4-mm cortical bone graft of donor mice was carefully dissected to remove the muscles without compromising the periosteum and then inserted into the host mice with the same size of the segmental defect. The bone graft was stabilized by a 25-gauge stainless steel needle placed through intramedullary marrow cavity. Four groups of bone grafting were performed: AR^flox/Y^ donor to AR^flox/Y^ host (WT-to-WT), AR^-/Y^;Prrx1::Cre donor to AR^-/Y^;Prrx1::Cre host (KO-to-KO), AR^flox/Y^ donor to AR^-/Y^;Prrx1::Cre host (WT-to-KO), and AR^-/Y^;Prrx1::Cre donor to AR^-/Y^ host (KO-to-WT). The grafted femurs were processed for histological and micro-CT analyses at the end time points of the experiments.

### Histological and histomorphometric analysis

The grafted femurs were harvested, fixed in 10% neutral buffered formalin (Sigma-Aldrich), decalcified in 10% EDTA (Sigma-Aldrich). For histological analyses, tissues were fixed in 4% formaldehyde (Sigma-Aldrich) in PBS overnight. Postnatal tissues were decalcified in Surgipath Decalcifier II (Leica Biosystems]), then embedded in paraffin. Sections were cut to a thickness of 5 μm, dewaxed, rehydrated with a graded ethanol series, and stained with Alcian Blue (Sigma-Aldrich)/Hematoxylin G (Sigma-Aldrich) stain and Safranin O (Sigma-Aldrich)/Fast Green (Sigma-Aldrich) stain and Goldner Trichrome (EMS 26386) as previously described [75]. Histomorphometric analyses were performed as previously described [76]. By using Osteometrics^TM^ software, the area of bone, cartilage and fibrotic tissue formation on the host and graft side were determined. A hypothetical line was drawn in the middle of the distal or proximal junctions between the graft and host bone to separate the callus into the graft side callus and the host side callus. Areas of bone, cartilage and fibrotic tissue were traced in a computer program and percent area was used for analyses. At least three non-consecutive sections were used for histomorphometric analyses and a mean of three represents one sample. At least eight samples were included in each group of transplantation. The mean from eight samples was used in statistical analyses to determine the composition of the callus on the host side and graft side.

### Immunohistochemistry, immunofluorescence and X-gal staining

For immunohistochemistry, sections (5-μm thick) were dewaxed, rehydrated using a graded ethanol series, and subjected to antigen retrieval. Thereafter, sections were first incubated overnight at 4 °C with rabbit polyclonal anti-mouse AR primary antibody (Santa Cruz: sc-7305), diluted 1:100, and then with horseradish peroxidase (HRP)-conjugated anti-rabbit secondary antibody (Dako: K5007). Sections were then incubated with DAB substrate (Dako), dehydrated, and coverslip-mounted. For immunofluorescence, sections (5-μm thick) were incubated with 5% horse serum for 30 min to block unspecific antibody binding, then incubated overnight at 4 °C in a mixture of anti-AR (Santa Cruz: sc-7305) and anti-Prrx1 (Santa Cruz: sc-293386) primary antibodies, diluted 1:100 in 1% horse serum/TBST (Tris-buffered saline containing 0.1% Tween-20). Thereafter, sections were incubated for 2 h at room temperature in the dark with Texas Red (Thermo Fisher Scientific)-conjugated (AR) and fluorescein isothiocyanate (Thermo Fisher Scientific)-conjugated (Prrx1) species-appropriate secondary antibodies, both diluted 1:100 in 1% horse serum. Sections were counterstained with 4’,6-diamidino-2-phenylindole (Thermo Fisher Scientific). For X-gal staining, tissues were fixed, decalcified and embedded in optimal cutting temperature medium (see the University of Rochester Medical Center protocol). Frozen tissue sections were cut into 10-μm sections, stained by immersion in 50 mg/ml X-gal (Sigma-Aldrich) staining solution overnight at room temperature in the dark, and counterstained with Nuclear Fast Red (Sigma-Aldrich). Images were acquired with a Nikon microscope.

### Isolation of periosteum-derived cells

The procedure for isolating periosteal cells was established as previously described [29]. Briefly, surrounding soft tissues (skin, muscle and epiphyseal cartilage) were removed from each skeletal element prior to isolation. Bone grafts were collected at day 5 post transplantation. Bone marrow was removed by repeatedly flushing of the marrow cavities with a serum-free α-MEM medium. Tissues attached to the periosteal surface of collected bone grafts were scraped off and pooled in a petri dish. The released cells were collected by centrifugation, resuspended, and filtered through a 70-μm cell strainer. Cells were cultured in αMEM containing 20% fetal bovine serum (FBS; Invitrogen), 100 U/ml penicillin/streptomycin (Invitrogen), and 2 mM L-glutamine (Invitrogen). Cells collected from the second and third passages were used for RNA extraction or fluorescence-activated cell sorting (FACS) analyses for stem cell surface markers.

### Cell culture

Validation for cell sorting and transfection was done with D1 cells from ATCC (ATCC^®^ CRL-12424^™^), a commercially available mouse bone marrow precursor cell line. These cells were not independently authenticated since they were obtained directly from ATCC. Cells were tested for mycoplasma using the PlasmoTestTM – Mycoplasma Detection Kit from InvivoGen (Cat # rep-pt2) and PCR analysis. All cells were confirmed to be free of mycoplasma.

### Fluorescence-activated cell sorting (FACS) analyses

Cell surface markers analysis was performed using phycoerythrin-conjugated antibodies against Sca-1 (eBioscience 11:5781-81), CD29 (eBioscience 12:0291-81), CD105 (eBioscience 12:1051-82), CD140, (eBioscience 14:1401-82) CD11b (eBioscience 12:0112-81), CD34 (eBioscience 14:0341-81) and CD45 (eBioscience 14:0451-81), followed by flow cytometry using a FACSAria II system (BD Biosciences, San Jose, CA, USA) in the CGMH Core facility. Voltages and gates were set based on unstained periosteum samples from Cre-negative animals.

### Multilineage differentiation analyses

For adipogenesis assays, cells were cultured in αMEM containing 15% FBS, 100 U/ml penicillin/streptomycin, 2 mM L-glutamine, 10^–6^ M dexamethasone (TOCRIS), 50 μM indomethacin (Sigma), and 100 μg/ml insulin (Invitrogen). The medium was replaced every 2–3 days, and after 21 days, cells were stained with Oil Red O (Sigma). For osteogenesis assays, cells were cultured with fresh osteogenic differentiation media containing 10 mM β-glycerolphosphate (Sigma) and 50 μg ascorbic acid (Sigma). The medium was replaced every 2–3 days, and after 28 days, cells were stained with Alizarin Red S (Sigma) or Von Kossa (Abcam). For chondrogenic assays, 7.1 × 10^4^ cells were resuspended in a 10-μl droplet and plated as a micromass in the center of a 48-well plate. Cells were incubated for 1.5 h at 37 °C in a humidified 5% CO_2_ incubator. Chondrogenic differentiation medium containing high-glucose DMEM supplemented with 1% ITS-Premix (Thermo Fisher Scientific), L-ascorbic acid-2-phosphate (Sigma) (0.1 mM), dexamethasone (Sigma) (1 × 10^7^ M), proline (Sigma) (400 mg/ml) and BMP-2 (Sigma) (100 ng/ml) was added onto the micromass droplet. The medium was replaced every 2–3 days, and after 9 days, micromass pellets were collected for Alcian Blue staining.

### CFU-F assay

Colony-forming unit fibroblast (CFU-f) assays were performed using primary periosteal cells isolated from 8-week-old mice. Mononucleated cells were plated in 6-well plates at a density of 5 × 10^5^ cells per well. Cells were fixed with methanol and washed with double-distilled H_2_O. After culturing for 14 days, cells were stained with 0.1% methylene blue (RHD) to visualize and facilitate the counting of CFU-f colonies (>20 cells colony).

### Semi-quantitative and quantitative RT-PCR, and osteogenesis PCR arrays

Total RNA was extracted from TM4, D1, AR^flox/Y^ and AR^-/Y^;Prrx1::Cre PDCs cells using the TRIzol reagent (Invitrogen) according to the manufacturer’s instructions. cDNA was reversed transcribed from 2 μg mRNA using a Promega RT-PCR kit. For semi-quantitative analyses, PCR was performed on an Applied Biosystems GeneAmp PCR system Veriti 96-well Thermal cycler (27–35 cycles), and band intensities were compared during the linear portion of the amplification cycle. Quantitative PCR was performed on an Applied Biosystems 7500 Real-time PCR detection system using cDNA-specific gene primers and SYBR green master mix (Applied Biosystems). Gene expression levels were compared using the comparative cycle threshold method, with β-actin as input control.

For osteogenesis, microarray analyses were performed on an Applied Biosystems 7500 Real-time PCR detection system using a mouse osteogenesis PCR array kit (Qiagen). The mouse osteogenesis RT² Profiler PCR Array profiles the expression of 84 genes related to osteogenic differentiation. This array contains genes that function in the development of the skeletal system as well as in bone mineral metabolism, and includes growth factors and genes that mediate osteogenesis and related cell growth, proliferation, and differentiation processes. Also represented are ECM molecules and cell adhesion molecules involved in bone development.

### Cell migration assay

Primary PDCs were isolated from AR^flox/Y^ and AR^-/Y^;Prrx1::Cre mice. The AR in PDCs from AR^flox/Y^ mice was knocked down using AR-targeting siRNA. D1 cells were transfected with Flag or Flag-tagged AR. All cells were cultured in αMEM containing 15% FBS, 100 U/ml penicillin/streptomycin, 2 mM L-glutamine on Oris^TM^ cell migration plates containing cell seeding stoppers. The cells were allowed to attach and spread for 16–20 h prior to manual removal of the stoppers. The cells were washed and then incubated for 30 min in 100 μl of αMEM medium containing 50 μM Cell Tracker Blue (Invitrogen). The cells were washed, and the medium was replaced with αMEM containing 15% FBS, with or without 2 μg collagen I (R&D), 30 nM TC-I 15 (α2β1 integrin inhibitor; TOCRIS) or 10 μg/ml CD49 antibody (Becton Dickinson). Images were captured on an Acumen explorer using a 488-nm laser and >655-nm filter. A square around the cell-free zone—the migration area—was defined. For siRNA assay, si-integrinβ1 #16412, si-FAK #14083–ON-TARGETplus SMARTpool-siRNA from Dharmacon, a rectangle encompassing the confluent cell monolayer from the same well-defined migration region. Cells were gated using mean intensity measurements. D1 cells (multipotent mouse bone marrow stromal precursor) were transfected with PSG5 or PSG5-AR. All cells were cultured in αMEM containing 15% FBS, 100 U/ml penicillin/streptomycin, 2 mM L-glutamine on Millicell Single Well Hanging Inserts cell migration plates (Millipore: PIEP 12R 48) containing cell seeding stoppers. Prepare PSG5, PSG5-AR cell suspension containing 0.5–1.0 × 10^6^ cells/ml in serum-free media. Add 500 µl of media containing 10% FBS to the lower well of the migration plate. Add 300 µl of the cell suspension solution to the inside of each insert. Incubate for 48 h in a cell culture incubator. Carefully aspirate the media from the inside of the insert. Wet the ends of three cotton-tipped swabs and gently swab the interior of the inserts to remove non-migratory cells. Ta6ke care not to puncture the polycarbonate membrane. Be sure to remove cells on the inside perimeter of the insert. Transfer the insert to a clean well containing 400 µl of Cell Stain Solution (10% giemsa stain: Merck 654833) and incubate for 10 min at room temperature. Gently wash the stained inserts several times in a beaker of water. Allow the inserts to air dry. Count migratory cells with a light microscope under a high magnification objective, with at least three individual fields per insert.

### Immunoprecipitation

Cells were lysed in lysis buffer (25 mM Tris-HCl pH 7.4, 150 mM NaCl, 1 mM EDTA, 1% NP-40, 5% glycerol, 1 mM phenylmethylsulfonyl fluoride (PMSF), 1 mM Na_3_VO_4_, 1 mM NaF, and 1 µg/ml aprotinin). Cell lysates containing an equal amount of protein (1.2 mg) were incubated with 4 μg anti-AR or anti-FAK antibody at 4 °C overnight. Protein–protein interactions were studied by performing immunoprecipitation using a Catch and Release v2.0 reversible immunoprecipitation system (Millipore Corporation), as per the manufacturer’s instructions. Thereafter, proteins in samples were resolved by sodium dodecyl sulfate–polyacrylamide gel electrophoresis (SDS–PAGE), transferred to PVDF membranes, and immunoblotted with anti-AR (Millipore PG-21 #06-680), anti-ARA55 (BD Biosciences #611164) or anti-FAK antibody (BD Biosciences #610088).

### Immunoblotting

Cell extracts were prepared in lysis buffer (50 mM Tris-HCl pH 6.8, 120 mM NaCl, 1 mM EDTA, 0.5% Nonidet P-40, 1 mM PMSF, 1 mM β-mercaptoethanol, 1 mM Na_3_VO_4_, 1 mM NaF, and 1 μg/ml aprotinin) for 30 min on ice, and centrifuged at 15,000 × g for 15 min to remove insoluble materials. Lysates were centrifuged at 14,000 rpm for 10 min, and the resulting supernatants were used for immunoblotting. Protein concentrations were determined using the Pierce protein assay reagent (Pierce, Rockford, IL, USA). Cell lysates containing equal amounts of protein (40 μg) were analyzed by SDS–PAGE. After transferring to Hybond-P membranes and blocking with 5% milk/0.1% TBST at room temperature for 60 min, membranes were incubated first with primary antibodies AR (Millipore PG-21 #06-680), FAK (BD Biosciences #610088), p-FAK(BD Biosciences #611722), ARA5 (BD Biosciences #6111645), Intergrin β1 (Cell Signaling #4706), GAPDH (Millipore #AB2302) overnight at 4 °C, and then with HRP-conjugated anti-mouse or anti-rabbit IgG secondary antibodies. Immunoreactive proteins were detected using an enhanced chemiluminescence detection kit (Millipore). Signals were analyzed and quantified using an LAS-3000 imaging system (Fujifilm, Tokyo, Japan).

### Combination therapy of testosterone and PDCs in mice with critical-size segmental defects

Eight-week-old AR^flox/Y^ or AR^-/Y^ male mice were used to generate a segmental defect of approximately 2.5 mm on the left femur of each mouse using a rotating blade with copious irrigation as we previously described [43]. Briefly, a 2.5-mm PPF/TCP scaffold loaded with phosphate-buffered saline (PBS; blank) or 100 μg testosterone (Sigma) was inserted to bridge the fracture. After the fracture, 1 × 10^6^ PDCs from AR^flox/Y^ and AR^-/Y^ mice were transplanted into segmental defected mice treated with PBS or testosterone (Sigma). A 25-gauge stainless steel needle was used as an intramedullary pin. The needle was drilled into the trochlear groove between the lateral and medial condyles to reach the femur marrow cavity. It was allowed to pass through the central channel of the scaffold and attached to the proximal end of the femur marrow cavity. Mice were given buprenorphine (0.1 mg/kg) for pain relief for 7 consecutive days, and then were returned to their home cages and allowed to move freely.

### Statistical analyses

Panels generally represent multiple independent experiments performed on different days with different mice. No blinding experiment or randomization-based analysis was used. Data are presented as mean ± SEM unless otherwise stated. Unpaired, two-tailed Student *t* tests were used for comparisons between two groups. For multiple comparisons, one-way ANOVA with Dunn’s post hoc test was applied. A *P* value less than 0.05 was considered significant. Each animal was assigned an identification number using the animal’s litter number in combination with the ear tag number. All data were normally distributed and had similar variations between groups. Data were analyzed by SPSS version 15 or SigmaStat statistics version 4.

### Study approval

We maintained all animals in the animal facility of the Chang Gung Memorial Hospital, Kaohsiung, Taiwan. The experimental protocols followed the Guide for the Care and Use of Laboratory Animal of the Institute of Laboratory Animal Resources, National Research Council, National Academy of Sciences, USA, and approved by the Institutional Animal Care and Use Committee of the Chang Gung Memorial Hospital, Kaohsiung, Taiwan.

## Supplementary information

Supplementary Figure 1

Supplementary Figure 2

Supplementary Figure 3

Supplementary Figure 4

Supplementary Table 1

Supplementary Table 2

## Data Availability

All data generated or analyzed during this study are included in this published article.
