# Supplementary figures and images for: Targeted activation of androgen receptor signaling in the periosteum improves bone fracture repair
Source: Cell Death Dis. 2022 Feb 8;13(2):123. doi: 10.1038/s41419-022-04595-1 (PMC8826926; doi:10.1038/s41419-022-04595-1)

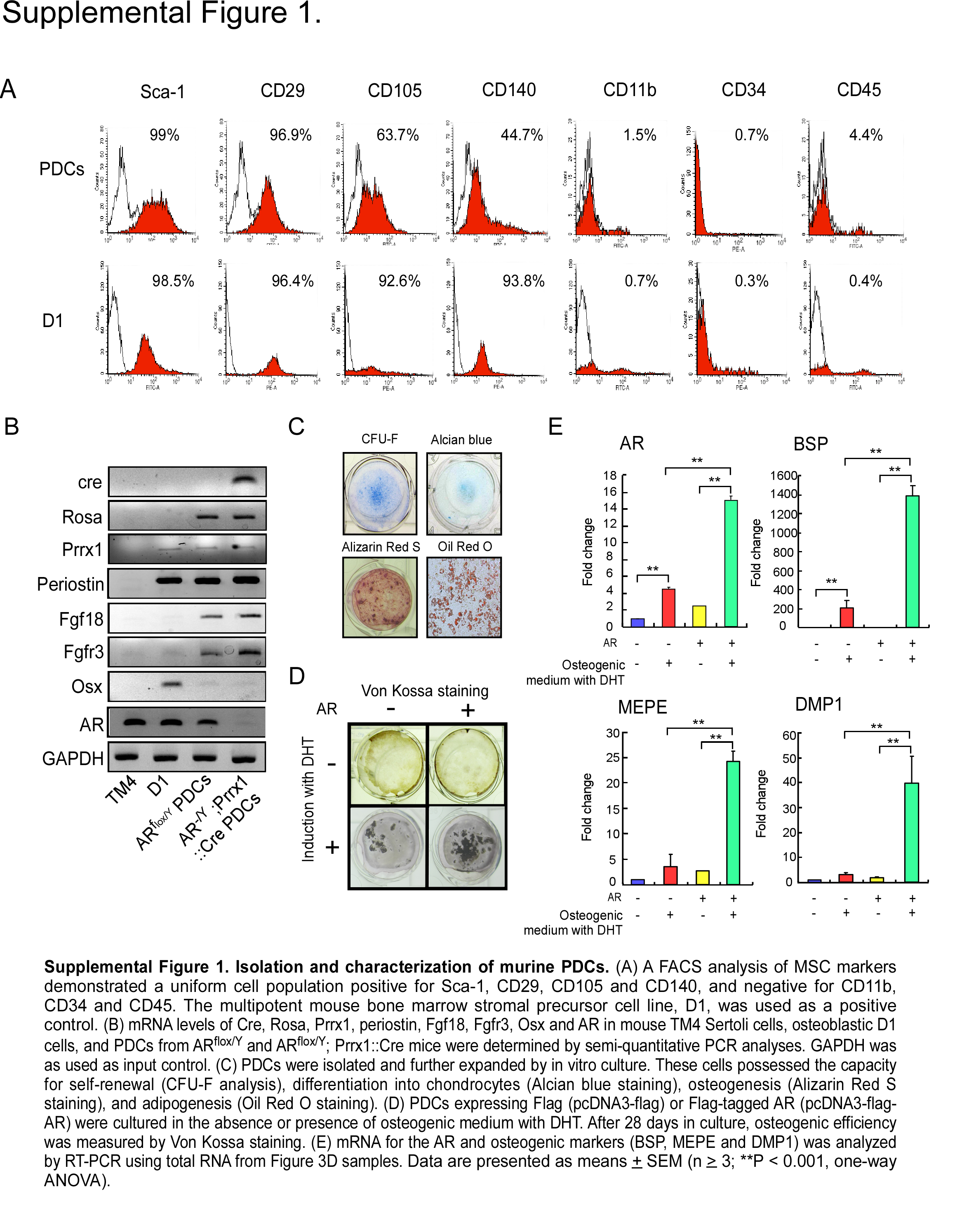

Supplement: Supplementary file 1 — Supplementary Figure 1 [file 41419_2022_4595_MOESM1_ESM.tif]

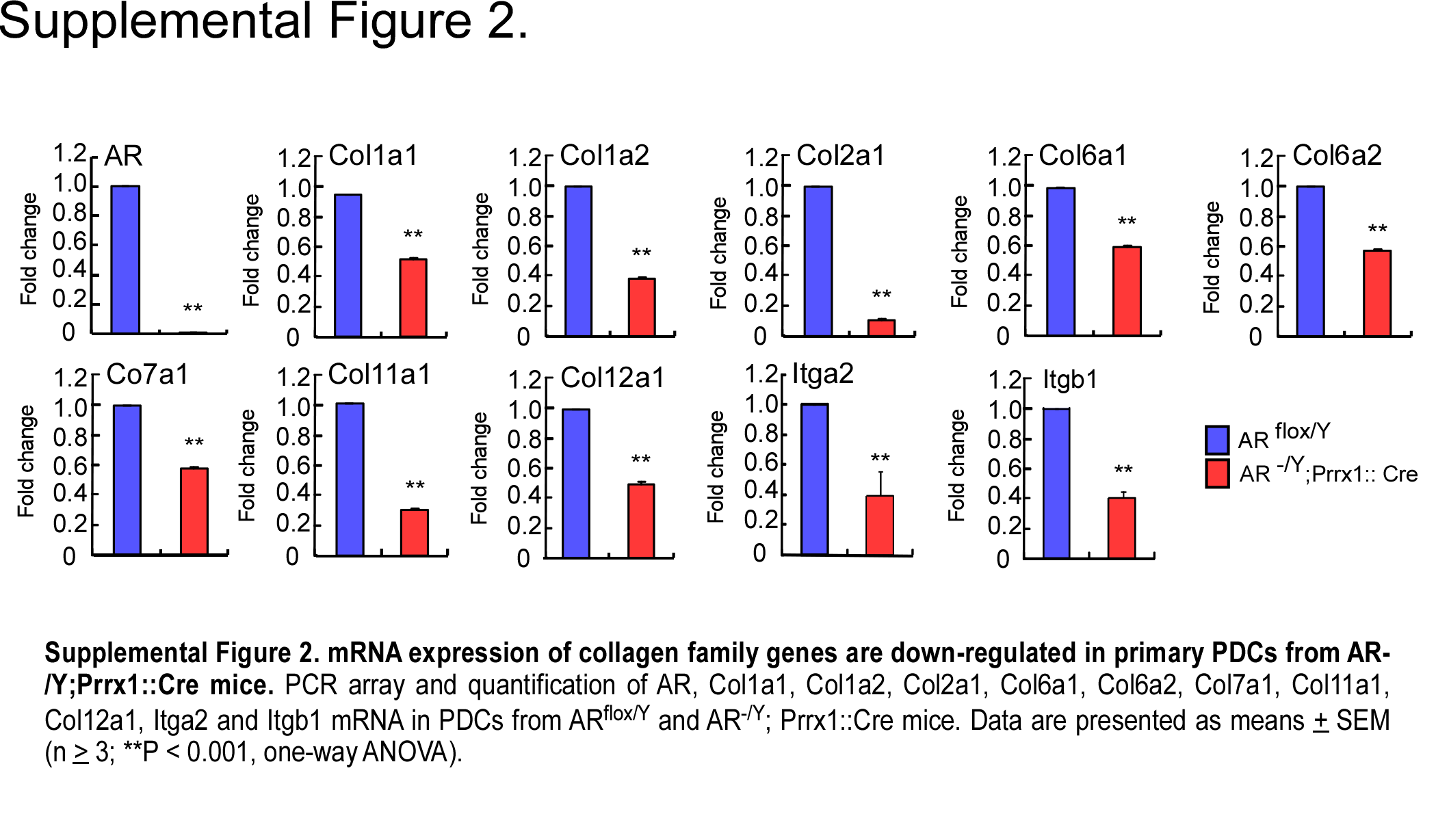

Supplement: Supplementary file 2 — Supplementary Figure 2 [file 41419_2022_4595_MOESM2_ESM.tif]

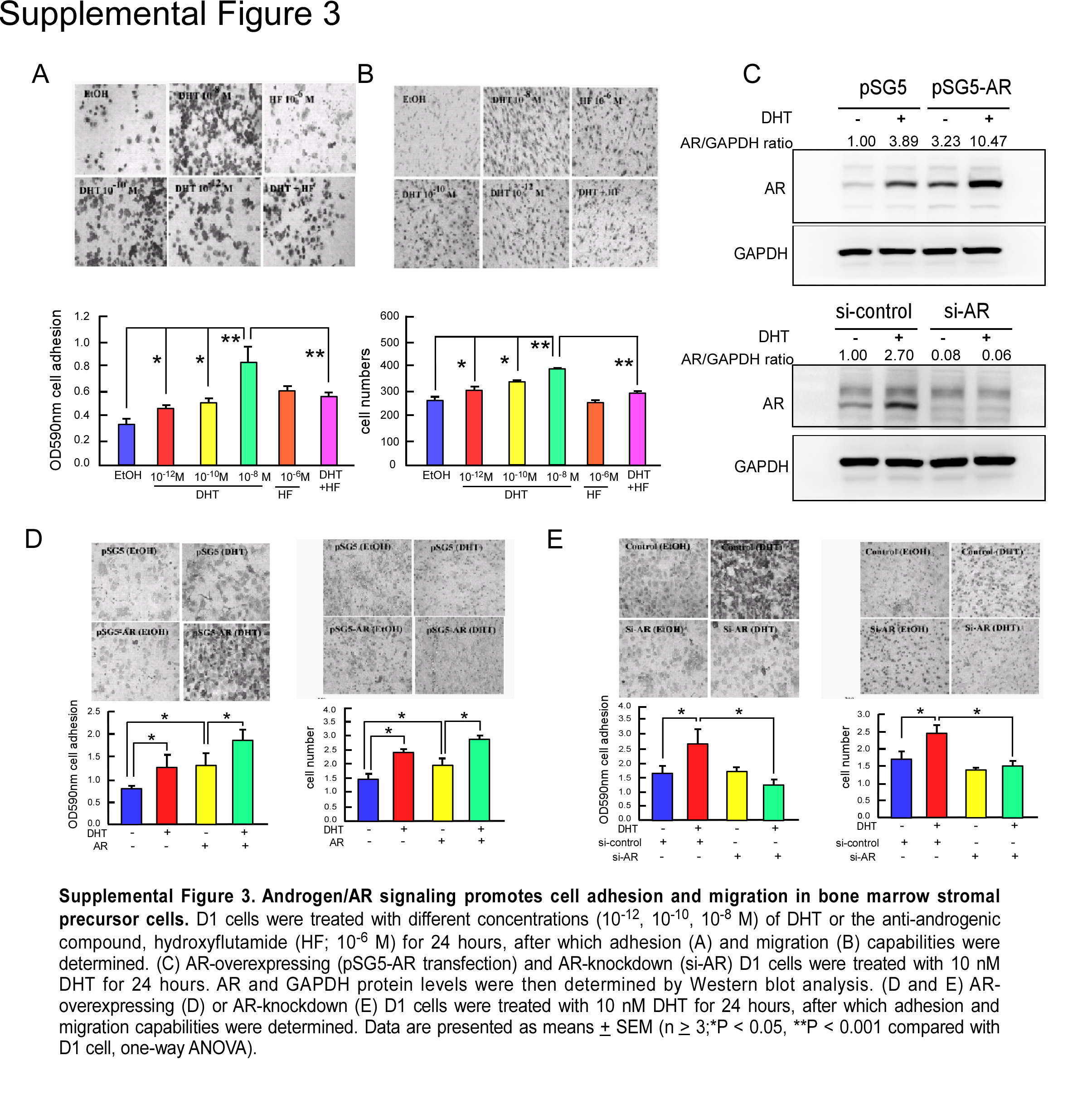

Supplement: Supplementary file 3 — Supplementary Figure 3 [file 41419_2022_4595_MOESM3_ESM.tif]

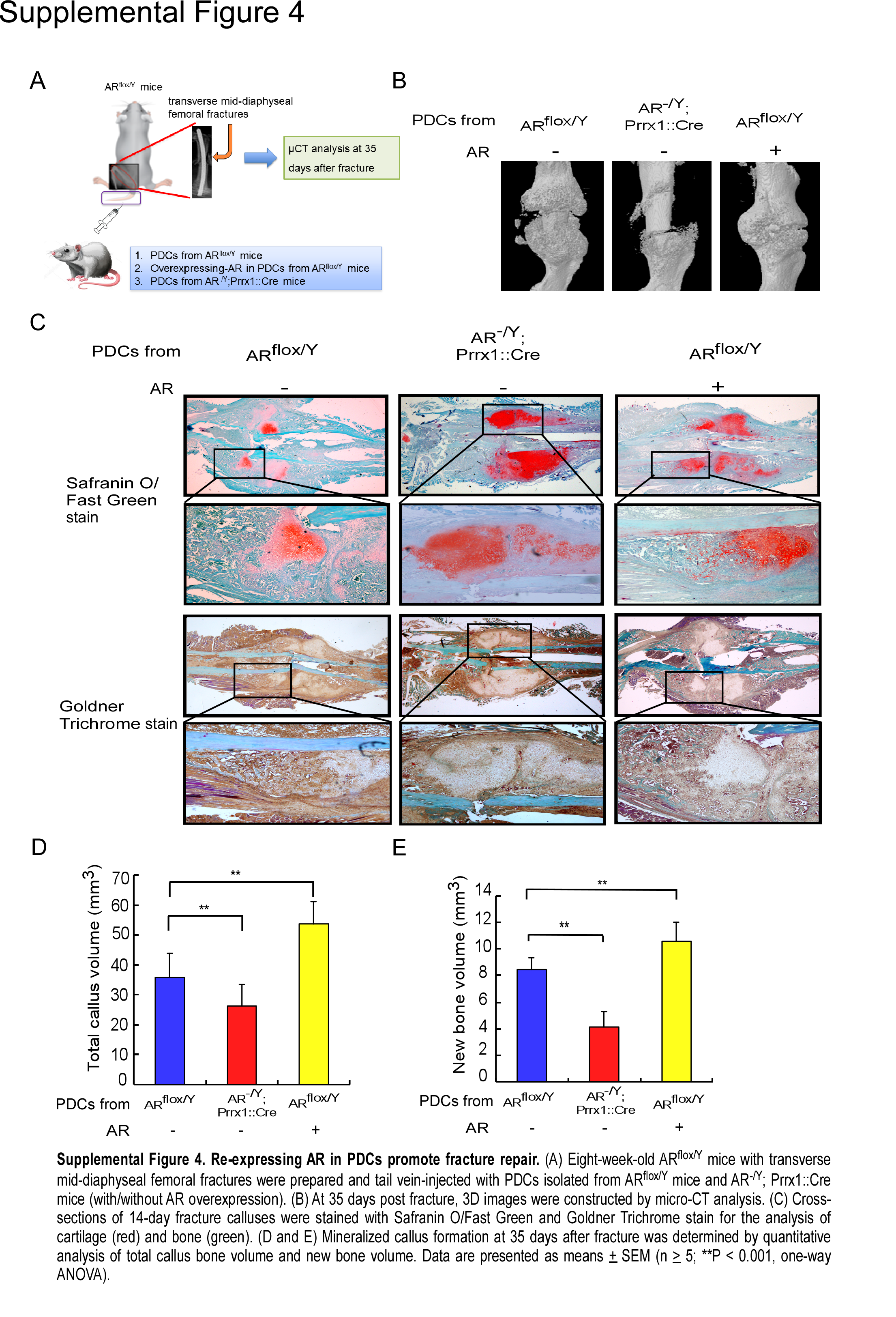

Supplement: Supplementary file 4 — Supplementary Figure 4 [file 41419_2022_4595_MOESM4_ESM.tif]
