# Supplementary material for: Targeted activation of androgen receptor signaling in the periosteum improves bone fracture repair
Source: Cell Death Dis. 2022 Feb 8;13(2):123. doi: 10.1038/s41419-022-04595-1 (PMC8826926; doi:10.1038/s41419-022-04595-1)
Supplement: Supplementary file 5 — Supplementary Table 1 [file 41419_2022_4595_MOESM5_ESM.doc]

| **Supplemental Table 1. Microarray analysis were differentially expressed in** | | **PDCs isolated from the ARflox/Y and the AR-/Y; Prrx1::Cre mice.** |
| --- | --- | --- |
| Category | | Gene symbol |
| Skeletal Development | Bone Mineralization | BSP, MEPE, DMP1 |
|  | Ossification | Sost, Ambn, Enam |
|  | Osteoclast Differentiation | Tnf |
| Cell Growth and Differentiation | Regulation of Cell Cycle | Fgfr3 |
|  | Growth Factors and Receptors | Gdf10, BMP6, BMP3, Fgfr3, CD36, Itgam |
|  | Cell Differentiation | Csf2, BMP6, AR, Twist1, Sox9 |
| Extracellular Matrix(ECM) Proteins | Collagens | Col1a1, Col1a2, Col2a1, Col6a1, Col6a2, Col7a1, Col11a1 |
|  | ECM Protease Inhibitors | Serpinh1, Col7a1 |
|  | ECM Proteases | Mmp8, Mmp9, Mmp10 |
| Cell Adhesion Molecules | Cell-cell Adhesion | Cdh11, BMP5 |
|  | Cell-matrix Adhesion | Itga2, Itga2b, Itga3, Itgb1 |
|  | Other Cell Adhesion Molecules | Comp, Col12a1, Col6a2, Col6a1 |

Blue color represents the genes the genes that decrease more than 2 folds in ARKO group; Red color represents the genes the genes that increase more than 2 folds in ARKO group
