# Supplementary material for: Targeted activation of androgen receptor signaling in the periosteum improves bone fracture repair
Source: Cell Death Dis. 2022 Feb 8;13(2):123. doi: 10.1038/s41419-022-04595-1 (PMC8826926; doi:10.1038/s41419-022-04595-1)
Supplement: Supplementary file 6 — Supplementary Table 2 [file 41419_2022_4595_MOESM6_ESM.docx]

**Supplemental Table 2 Primer sequences for quantitative RT-PCR**

| Gene | Primers (F=forward; R=reverse) |
| --- | --- |
| AR | F:GCTGCCTTGTTATCTAGCCT  R: AATGACCGCCATCTGGTCAT |
| Col1a1 | F:GAGAGCATGACCGATGGATTC  R:ACGCTGTTCTTGCAGTGATAG |
| Col1a2 | F:GCTGGTGTAATGGGTCCTCC  R:CGACCGGCATCTCCATTAGG |
| Col2a1 | F:GCA GAG ATG GAG AAC CTG GTA  R:AGC CTT CTC GTC ATA CCCT |
| Col6a1 | F:TGGGGAATGCATTTTACCAT  R:AAATCGTGGTCCCCAAGC |
| Col6a2 | F:TGCTCCGTGCTCCTGCTCTG  R:ATGGTGACGCTCTCCGAGGTG |
| Col7a1 | F:GATGACCCACGGACAGAGTT  R:ACTTCCCGTCTGTGATCAGG |
| Col11a1 | F:GACTATCCCCTCTTCAGAACTGTTAAC  R:CTTCTATCAAGTGGTTTCGTGGTTT |
| Col12a1 | F:TATTGTGTTCTTGACTGATGCCTCCTG  R:AGACTTGACCTCATCGCTGTATTGC |
| Itga2 | F:TTTCTGCAGCCTAAGGGCC  R:GGCAGCCACAGCAATATCATT |
| Itgb1 | F:CTCCAGAAGGTGGCTTTGATGC  R:GTGAAACCCAGCATCCGTGGAA |
